# Antimicrobial Activities of Natural Bioactive Polyphenols

**DOI:** 10.3390/pharmaceutics16060718

**Published:** 2024-05-27

**Authors:** Manas Kumar Mandal, Abraham J. Domb

**Affiliations:** The Alex Grass Center for Drug Design & Synthesis and the Center for Cannabis Research, School of Pharmacy, Institute of Drug Research, Faculty of Medicine, The Hebrew University of Jerusalem, Jerusalem 9112001, Israel; manas.mandal@mail.huji.ac.il

**Keywords:** bioactive polyphenols, classification source polyphenols, antimicrobial activities, health-benefited polyphenols

## Abstract

Secondary metabolites, polyphenols, are widespread in the entire kingdom of plants. They contain one or more hydroxyl groups that have a variety of biological functions in the natural environment. These uses include polyphenols in food, beauty products, dietary supplements, and medicinal products and have grown rapidly during the past 20 years. Antimicrobial polyphenols are described together with their sources, classes, and subclasses. Polyphenols are found in different sources, such as dark chocolate, olive oil, red wine, almonds, cashews, walnuts, berries, green tea, apples, artichokes, mushrooms, etc. Examples of benefits are antiallergic, antioxidant, anticancer agents, anti-inflammatory, antihypertensive, and antimicrobe properties. From these sources, different classes of polyphenols are helpful for the growth of internal functional systems of the human body, providing healthy fats, vitamins, and minerals, lowering the risk of cardiovascular diseases, improving brain health, and rebooting our cellular microbiome health by mitochondrial uncoupling. Among the various health benefits of polyphenols (curcumin, naringenin, quercetin, catechin, etc.) primarily different antimicrobial activities are discussed along with possible future applications. For polyphenols and antimicrobial agents to be proven safe, adverse health impacts must be substantiated by reliable scientific research as well as in vitro and in vivo clinical data. Future research may be influenced by this evaluation.

## 1. Introduction

Polyphenols are natural organic products having several hydroxyl groups. (-OH) is the aromatic ring largely found in fruits, vegetables, cereals, and beverages [1,2,3,4,5,6,7]. They generally fall under three main categories: phenolic acids, flavonoids, and non-flavonoids. These three are thought to have a variety of antimicrobial health advantages [8,9,10,11,12,13]. The need for healthy ingredients, nutritional supplements, and medicines has stimulated an interest in research involving the use of polyphenols as antimicrobial compounds. To deal with current difficulties that restrict the use of polyphenols, such as their quick elimination from the body, low water solubility, unstable acidic conditions, and their particle size, research is being undertaken. More than 8000 various types of polyphenols have been recognized to date [14,15,16,17]. Since the dawn of civilization, infectious microbes have posed a threat as a significant source of illness and mortality. The year 1928 marked the discovery of the first antibiotic penicillin, and then the 1930s saw development of sulfa medicines. These, and the use of different plant extracts, were the only effective treatments for infections. These treatments, however, had varying degrees of success in medical science [18,19,20]. Whereas antibiotics have served as an important part of treating infectious diseases brought on by fungal and bacterial infections for the last few decades, harmful and antibiotic-resistant bacteria have been seen more frequently in recent years. Almost 100,000 different plant species have been examined for the possibility of being used as medicines for the treatment of various diseases. According to 2007 WHO estimates, 25% of medicines on the market come from plants used in traditional medicine [21,22,23]. In addition to their lengthy history of therapeutic use, plant-based substances show favorable reception among patients and tolerances, making them appear to be a reliable source of antibacterial agents [24,25,26].

This review, a comprehensive overview of flavonoid structure, fundamental characteristics, and distribution, discusses the range of their antibacterial action as a potential alternative to traditional medications. We have examined newly discovered flavonoid compounds that exhibit strong antibacterial properties, and we have provided examples of flavonoids that exhibit reciprocal and combined consequences and also can be used in medicinal and food chemistry [27].

Various microbial agents are destroyed by bioactive polyphenols through their entry into cellular systems by rupturing the cell membrane by hydrophobic interactions as well as inhibiting enzyme activities, DNA gyrase, and RNA synthesis. As a result, foreign bodies are not capable of living in the human body and are not able to interrupt cellular functionality. Diets high in plant polyphenols for a substantial amount of time may safeguard against the onset of cancer, heart disease, diabetes, osteoporosis, and neurological illnesses, according to epidemiological research and related analyses [28,29,30,31,32]. Here, we discuss the antimicrobial effects of naturally occurring polyphenols, examining their potential impact on human health. Polyphenols, as secondary metabolites with a variety of important functions in plant physiology, offer promising health benefits for humans. These benefits include acting as antioxidant compounds, antiallergics, anti-inflammatories, anticancer agents, antihypertensives, and antimicrobial agents [33,34,35,36]. The antibacterial (Gram-positive and Gram-negative), antiviral, and antifungal properties of the most potent polyphenols are discussed in this research, with an emphasis mostly on the way they work as well as the structure–activity connections that have been studied. Bioactive polyphenols are obtained in plant-based foods. They are reviewed in this article, including in combination with their effective biological activities The antibacterial properties of polyphenols derived from various natural sources have been documented by extensive research. For instance, it has been demonstrated that polyphenols derived from green tea have antibacterial action against several bacterial species, such as *Escherichia coli*, *Staphylococcus aureus*, and *Streptococcus mutans* [37,38,39]. Similarly, it has been demonstrated that polyphenols derived from grape seeds have antibacterial activity against several bacterial species, including *Salmonella enteritidis*, *Listeria monocytogenes*, and *Bacillus cereus* [40,41,42,43]. It has also been discovered that polyphenols have antifungal properties. For example, it has been demonstrated that polyphenols derived from ginger have antifungal action against *Candida albicans*, a fungus that can infect people. Moreover, it has been demonstrated that polyphenols derived from turmeric exhibit antifungal action against *Aspergillus flavus*, a fungus that can contaminate food and lead to health issues [44,45,46,47]. There is variation in the distribution of phenolics in plants at tissues, cellular, and subatomic levels. Plant cell vacuoles contain soluble phenolics, whereas insoluble phenolics are located in the walls of the cells. Quercetin is one of the polyphenols that can be found in all plant products, including fruit, vegetables, cereals, tea, wine, and infusions. On the other hand, flavanones and isoflavones are unique to certain foods [48,49,50]. Foods typically contain complex polyphenol combinations. Plants with outer layers have concentrations of phenolics greater than those with interior layers. Rainfall, sunlight, soil type, and other edaphic and natural variables all have a significant impact on the polyphenolic constituents of food. Considerable ripeness influences the ratios and quantities of different types of polyphenols. Phenolic acid content has often been shown to decrease as anthocyanin contents rise during the ripening process. Numerous polyphenols, particularly phenolic acids, play a direct role in how plants react to various stresses. They have antibacterial qualities that help injured areas heal, and their amount may rise following an outbreak of infection [51,52,53].

Overall, polyphenols isolated from natural sources are attractive candidates in medicinal industries as well as in the food industry for the creation of novel antimicrobial medicines to fight infectious diseases due to the ability of their effective hydrophobicity to destroy the foreign microbial cell membrane as well as cell functionality [54,55,56,57].

## 2. Basic Information: Classifications, Molecular Structure, and Natural Sources

**Figure 1:**
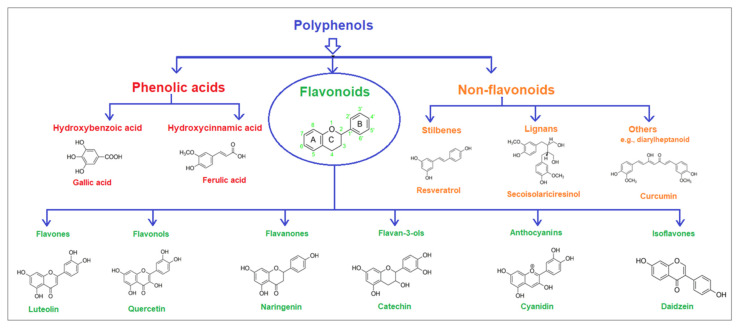
Classification of the polyphenols with examples based on structural elements that bind the rings one to another.

Polyphenolic acids are classified into two categories, benzoic acid and cinnamic acid derivatives. Several subclasses of non-flavonoids and flavonoids have general molecular structures: A, B, and C (Figure 1). A few examples of polyphenols are structurally represented here along with their classification [58,59].

### 2.1. Phenolic Acids

Foods include large amounts of phenolic acids. They are broken down into two classes: hydroxybenzoic acid and hydroxycinnamic acid derivatives. Except for some red fruits, black radish, and onions, which can have quantities of 10 mg/kg, the hydroxybenzoic acid level of food plants is typically modest. p-Coumaric, caffeic, ferulic, and sinapic acids make up the majority of the hydroxycinnamic acids, which are more prevalent than hydroxybenzoic acids [60,61,62,63]. Three primary marine macroalgae groups can also produce several antimicrobial polyphenols (mainly phenolic acids and flavonoids): brown algae (*Phaeophyta*), red algae (*Rhodophyta*), and green algae (*Chlorophyta*) [64].

### 2.2. Flavonoids

Naturally phenolic substances that are classified as flavonoids have a C_6_-C_3_-C_6_ carbon structure (phenyl benzopyran). The 2-phenyl-benzo-c-pyrane center of a flavonoid is composed of two benzene rings A and B connected by a heterocyclic pyran or pyrone ring C. Flavonoids can be divided into several subclasses according to their degree of unsaturation and oxidation, including flavones, isoflavones, flavonols, flavanols, flavanones, and flavanonols. Recently, a group of polyphenols known as flavonoids has received the most research attention. Two aromatic rings joined by three C-atoms to form an oxygenated heterocycle make up the general structure of this category (shown in Figure 1) [65]. Flavonoids are found in more than 4000 different forms, many of which are in charge of giving flowers, fruits, and leaves their eye-catching hues. Flavonoids can be classified into six subclasses (described in the Table 1) depending on the type of heterocycle involved as shown in Figure 1 [65,66].

#### Six Subclasses of Flavonoids

There are various classes of polyphenols based on their different poly hydroxyl group positions in the C_6_-C_3_-C_6_ carbon structure. In this section, six different classes of flavonoids are discussed, including their various antimicrobial activities. Due to the presence of polyphenolic compounds in their molecular structure, flavonoids, as a broad class of phytonutrients (chemicals found in plants), are classified as polyphenols. That indicates the structures of flavonoids contain several phenol units. “Polyphenol” derives from the words “poly” (many) and “phenol”, a chemical compound consisting of a six-carbon ring joined to a hydroxyl group (-OH). Among their many applications in biology, bioactive polyphenols are well known for their antioxidant, anticancer, and antimicrobial qualities.

The six groups of flavonoids are the following:

Flavonols (e.g., quercetin, kaempferol, etc.)—Found in onions, leeks, and broccoli.

Flavones (e.g., luteolin, apigenin, etc.)—Found in parsley, celery, and chamomile. 

Flavanones (e.g., hesperidin, naringenin, etc.)—Found in citrus fruits. 

Flavan-3-ols (e.g., catechin, epicatechin, etc.)—Found in green tea, chocolate, and grapes. 

Anthocyanins (e.g., cyanidin, delphinidin, etc.)—Found in red, purple, blue colors in many fruits and vegetables. 

Isoflavones (e.g., genistein, daidzein, etc.)—Found in soy products and other legumes.

Although all of these classes have a similar phenolic structure, they are divided into distinct six classes due to variations in the number of hydroxyl groups (-OH) attached and the positioning of carbon atoms, accordingly categorizing them into different classes of flavonoids. Their antimicrobial properties and effectiveness as antioxidants, which are essential for shielding human beings from oxidative damage and associated illnesses, are bolstered by their polyphenolic content. Henceforth, flavonoids having different numbers of –OH groups, as well as different positions in the phenyl benzopyran molecular structures of the corresponding flavonoids, provide different new classes and subclasses of polyphenols and are described with examples in tabular form (Table 1).

### 2.3. Non-Flavonoids

Among the non-flavonoids, two major classes (stilbenes and lignans) are considered in this section. Other sections are also important, since naturally occurring polyphenols (curcumin) have good antimicrobial characteristics [67]. Although non-flavonoids have lower antioxidant activity than flavonoids, as previously documented, several studies are nevertheless worthwhile to mention. Certain phenolic acids, such as ferulic, caffeic, and gallic acids, demonstrate antibacterial action against both Gram-negative (*E. coli* and *Pseudomonas aeruginosa*) and Gram-positive (*S. aureus* and *L. monocytogenes*) bacteria. It was discovered that these substances are more effective than common antibiotics like gentamicin and streptomycin against the bacteria. On the other hand, chlorogenic acid was ineffective against Gram-positive bacteria [67,68,69].

Stilbenes: Two Ph-moieties are joined by a two “C” methylene bridges in stilbenes. These are rather rare in human nutrition. Most stilbenes in vegetables serve as phytoalexins, which are compounds that are only generated in response to infection or injury. Resveratrol (3,4′,5-trihydroxystilbene), a naturally occurring stilbene that is mostly abundant in grapes, is one of the most well-studied polyphenol stilbenes. Red wine, a grape-based drink, has a sizable portion of resveratrol [70,71].

Lignans: The 2,3-dibenzyl-butane moiety of lignans, which are diphenolic substances, is created when two cinnamic acid residues are dimerized. Secoisolariciresinol is one of the lignans which acts as a phytoestrogen. The most abundant nutritional resource is linseed, which has significant amounts of matairesinol and secoisolariciresinol. Antimicrobial activity against Gram-positive (*S. aureus*, *S. epidermidis*, etc.) and Gram-negative (*Pseudomonas aeruginosa*, *Escherichia coli*, etc.) bacteria at low temperatures is also demonstrated by the presence of methoxy and phenolic hydroxyl groups in the lignans [72,73].

## 3. Activities as Antimicrobial Agents of Several Polyphenols

Salicylic acid suppresses the growth of various fungi and bacteria, including *Propionibacterium acnes*, linked to acne development. It disrupts bacterial membranes and hinders their metabolism [74]. Gallic acid/ellagic acid/4-hydroxybenzoic acid kills the microbial agents by interfering with cell walls, and metabolic processes disrupt enzyme activity, effectively combating bacteria, fungi, etc. p-coumaric acid is the main antibacterial component in alkaline hydrolysates compared to caffeic and ferulic acid while hydroxycinnamic acids also exhibit strong antibacterial effects. Particularly, p-coumaric acid, with IC_50_ ranging from 200–400 µg/mL, showed a stronger effect against various enterobacteria strains [75]. Apigenin demonstrates notable antibacterial and antibiotic-synergistic action toward oral infections. Significant growth inhibition of bacteria and fungi has been achieved by luteolin, baicalein, and tangetin as observed in earlier studies. Luteolin is a more effective antimicrobial agent through nucleic acid and protein synthesis inhibition, bacterial cell membrane impairment, cell morphological modification, and biofilm inhibition [76,77,78]. Algae like *Jania rubens* red seaweed [79] inhibited the growth of several bacteria, fungi, and viruses and, in addition, microbial cell growth is prevented by quercetin, rutin, morin, and myricetin. Flavonols like galangin and kaempferol, which is frequently present in propolis samples, have inhibitory effects against the aspergilli *Aspergillus tamarii*, *Aspergillus flavus*, *Cladosporium sphaerospermum*, *Penicillium digitatum*, and *Penicillium italicum* [20,80,81]. Naringenin and naringin treatments decrease lipid peroxidation and elevate antioxidant levels in rats. The pharmacological effects of hesperidin and eriodictyol include antibacterial and antifungal properties were observed and the antifungal and antibacterial activity of eriodictyol was high [82,83]. Catechin and arbutin restrict the growth of *Salmonella typhimurium.* ATCC 13311 and phloretin and phlioridzin inhibit fungi and bacterial species through decreased lymphatic node metastases and kill the microorganisms [3,84]. The antimicrobial properties of pelargonidin, delphinidin, cyanidin chloride, cyanidin 3-glucoside, malvidin, petunidin, and phenolic extracts from various berries were tested against *Lactobacillus* species and other intestinal bacteria [85]. The growth of *Listeria monocytogenes*, *Staphylococcus aureus*, *Bacillus subtilis*, *Enterococcus faecalis*, and yeast strains (*Debaryomyceshansenii*, *Trichosporoncutaneum*, etc.) was inhibited by anthocyanins [86,87,88]. *C. albicans* and *T. rubrum* are affected by daidzein and genistein. Glycitein shows effective antifungal as well as antibacterial properties [89,90]. In experiments using antimicrobial agents, strains susceptible to resveratrol (50 µg/mL) and curcumin (20 µg/mL) show sub-MIC levels lowering evaluated virulence factors by at least 50% (IC_50_). In the majority of isolations (57–94%), hemagglutination and hemolysin activities were 50% reduced. Protease and biofilm IC50 values were observed in 6.5–17.8% of isolates utilizing a methicillin-resistant strain of *Staphylococcus aureus* [91].

**Table 1:** Description of sources and different antimicrobial activity of corresponding polyphenols with IC50 values.

Class/Subclass of Polyphenols	Sources	Polyphenols	IC50 Values	Inhibition Target	Ref.
Hydrobenzoic acid	Red huckleberries, coriander, black radish, garden onions, etc.	Salicylic acid (SA)	5 mM	*Fusarium oxysporum*	[92]
		Gallic acid (GA)	1600 μg/mL	*Staphylococcus aureus*	[93]
		Ellagic acid (EA)	1 mM	*Helicobacter pylori*	[94]
		4-Hydroxybenzoic acid	926 μg/mL	*Staphylococcus aureus*	[95]
Hydroxycinnamic acids	Fruits, vegetables, and drinks (tea, wine, and coffee).	p-Coumaric acid	652 ± 3.3 μM	*B. subtilis*	[96]
		Caffeic acid	334 μM	*Y. enterocolitica*	[97]
		Ferulic acid	700 ± 3.4μM	*B subtilis*	[96]
Flavones	Leaves, flowers, and fruits including celery, parsley, red peppers, chamomile, mint, and Ginkgo biloba.	Luteolin	50 μM	*Helicobacter pylori*	[98]
		Apigenin	9.59 ± 0.54 mM	*S. aureus*	[99]
		Baicalein	25.86 μg/mL	*S. aureus*	[100]
Flavonols	Onions, kale, lettuce, tomatoes, apples, grapes, berries, tea, and red wine. In addition, other sources are algae like *Jania rubens* red seaweed.	Quercetin	65± 5 μM	*S. aureus* NCTC 5655 strain	[101]
		Morin	50 μM	*Vibrio cholerae*	[102]
		Myricetin	46.2 μM	*Escherichia coli*	[103]
		Rutin	357.8 ± 15.5 µg/mL	*F. oxysporum* f. sp. *vasinfectum*	[104]
		Kaempferol	50 ± 2 μM 22 ± 2 μM	*P. aeruginosa* *S. aureus* PriA	[105]
		Galangin	65 ± 5 µM	*Staphylococcus aureus*	[106]
Flavanones	Naringenin in grapefruit, tomatoes, mint, and citrus fruits and eriodictyol in lemons.	Naringenin	6.8 ± 0.4 μM	Viral replication	[107]
		Naringin	62.5 μg/mL	*S. aureus*	[108]
		Hesperidin	125 μg/mL	*Staphylococcus aureus*	[83]
		Eriodictyol	2.229 ± 0.014 μg/mL	*Staphylococcus aureus*	[109]
Flavan-3-ols	Important bioactives in tea, pome fruits, berries, cocoa-derived products, nuts, and other foods.	Catechin	5.65 µg/mL	*M. luteus*, *B. subtilis*, and *S. aureus*	[110]
		Arbutin	200 μg/mL	Human melanoma cell line (Fema-x)	[111]
		Phloretin	671.76 ± 9.03 µg/mL	*E. coli*	[112]
		Phlioridzin	5.1 ± 1.2 µg/mL	*Trichophyton violaceum*	[113]
		Proanthocyanidin	312 µg/mL	*Staphylococcus aureus* ATCC 25923	[114]
Anthocyanins	Red and purple berries, grapes, apples, plums, cabbage, and natural colorants.	Cyanidin	21.91 µg/mL	*Staphylococcus aureus*	[115]
		Malvidin	4 mg/mL	*B. cereus* ATCC 11778	[116]
		Delphindin	12.5 mg/mL	*Vibrio parahaemolyticus*	[116]
		Petunidin	30.78 ± 1.17 μM	*Saccharomyces cerevisiae*	[117]
		Pelagonidin	3.02 µg/mL	Staphylococcus aureus ATCC 29213	[118]
Isoflavones	Soy and its products, as well as legume seeds (lentils, beans, and peas).	Daidzein	15.1 μM	*E. coli*	[119]
		Glycitein	7.49–10.46 μM	*E. limosum*	[120]
		Genistein	281 ± 29 μM	SW480 cell	[121]
Non-flavonoids	Grapes, apples, blueberries, plums, peanuts, lentils, wheat, algae, some vegetables (carrots, asparagus, garlic), and curcumin in turmeric.	Resveratrol	16.23 µg/mL	*Leishmania major*	[122]
		Curcumin	13.67 μM	Anti-ZIKV (strain PAN2016)	[123]

## 4. Microbial (Bacteria, Fungi, Viruses) Species Sensitive to Polyphenols

One major group of bacterial species is multidrug resistant, posing a special risk to people who work in nursing homes and hospitals. Several microbes (e.g., *Staphylococcus aureus*, *Escherichia coli*, *Pseudomonas aeruginosa*, *Acinetobacter* sp., *Proteus* sp., *Micrococcus* sp., *Klebsiella pneumonia*, *Staphylococcus epidermidis*, *Bacillus subtilis*) are very much responsible for problems in our lives. These types of microbes are resisted by polyphenolic compounds (phenolic acids, flavonoids, and non-flavonoids) like catechin, phenolic acid, cinnamic acid, ferulic acid, sinapic acid, p-coumaric acid, epigallocatechin gallate, daidzein, resveratrol, curcumin, etc.

Microbes like *C. famata*, *C. utilis*, *C. albicans*, and *S. cerevisiae* are types of fungi that also have been restricted using broth dilution and agar disk diffusion methods [124]. Baicalein was shown by Chen et al. to inhibit replication with cell adhesion in *S. aureus* [125]. Since EGCG is capable of binding to porins, this is likely how it influences the ability to damage the exterior cell membrane of Gram-negative bacteria through porin pores. Several flavonoids successfully killed microbes like *Escherichia coli* by forming complexes with extracellular and dissolved proteins [126]. Some viruses destroy our lives easily. Among these, the most common viral pathogens are the influenza virus, rhinovirus, adenovirus, coronavirus, and respiratory syncytial virus (RSV), which are very much responsible for the above. In the presence of polyphenols like resveratrol, luteolin, ellagic acid, cyanidin, etc., harmful viruses like hepatitis B, influenza, etc., can be destroyed and save our lives. Using these more important polyphenolic compounds can protect from microorganisms like bacteria, fungi, viruses, etc. [127,128,129,130].

In addition, several profound antimicrobial properties of polyphenols in wound healing are discussed in this section. A wound or injury to the skin can be caused by several factors, such as heat damage, trauma, or long-term ulcerations brought on by diseases like diabetes mellitus. Healing is hampered by chronic wounds, which make treatment more difficult, lowering patient satisfaction and increasing expenses. Such wounds are quite prone to becoming infected. Novel antimicrobial polyphenolic systems are being investigated in light of the growing antibiotic resistance in conventional therapy. Although stability concerns may restrict the effectiveness of polyphenols as alternatives to antibiotics for the treatment of chronic wounds, they still show potential [51]. To accomplish this, developments in efficiency by delivering bioactive polyphenols as naturally occurring antibacterial agents aim to stabilize their use for complex wounds while improving wound healing. *S. aureus* and methicillin-resistant *S. aureus* are the primary microorganisms identified in the early stages of chronic wounds, while *E. coli* and other pathogens are found later as the disease progresses. Polyphenolic substances that promote wound healing include kaempferol, chlorogenic acid, resveratrol, and ferulic acid. Tannic acid is a useful substance for treating wounds because of several advantageous qualities. Nurettin Sahiner et al., developed an antioxidant antimicrobial hydrogel against various pathogens for chronic wounds. A recent study confirms the wound-healing effects of tannic acid in both lab and animal tests, demonstrating its ability to stimulate hair follicles and accelerate skin regrowth. This suggests tannic acid could be valuable in natural wound care materials. The results demonstrate that whereas pectin- and nanoparticulate-based systems improve resveratrol distribution by resolving its solubility and bioavailability issues, microencapsulation of polyphenols boosts cutaneous wound healing with curcumin. Resveratrol in capsules exhibits more antimicrobial action compared to its natural version [52,53,131].

## 5. Possible Antimicrobial Mechanisms (Inhibition Pathways)

**Figure 2:**
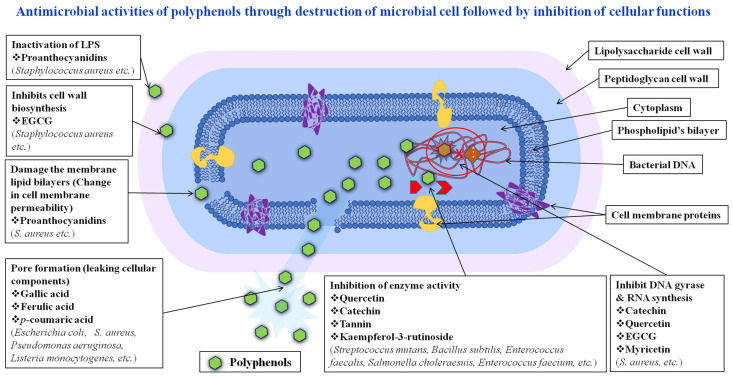
Schematic diagram of antimicrobial effect of natural polyphenols through inhibition of intracellular functions.

Polyphenols have a variety of antimicrobial properties to counter bacteria, fungi, and viruses. They have the ability to cause quorum sensing to interfere with and break cell membranes, inhibit enzymes, produce reactive oxygen species (ROS), chelate metal ions, alter the host immunological response, and prevent viral entry and reproduction [132]. Through these processes, polyphenols are able to kill the microbial species. While enzyme inhibition can cause metabolic pathways to be disrupted, disruption of cell membranes has the potential to cause cell death. Microbiological elements may sustain oxidative damage from reactive oxygen species, which can result in cell death. Additionally, polyphenols can disrupt quorum-sensing commands, which can impact gene expression and the development of biofilms. Additionally, by modifying the host immune response, they can strengthen defenses against microbiological infections. The particular mechanisms at play may differ based on the objective microorganism, the molecular structure of secondary metabolites, and the surrounding circumstances. Part of the antibacterial capacity of polyphenols comes from their ability to break down microbe cell membranes [26,133]. They cause cellular loss and death through their interactions via lipid bilayers, membrane permeability, and disruption of protein chains in the membrane that releases reactive oxygen species, damaging the integrity and functionality of membranes and ultimately causing cell lysis. This process may cause the microorganisms to lose their internal contents and disrupt their biological functions. Studies indicated that polyphenols, which are effective antifungal, antiviral, and antibacterial agents, have emerged as a potent subject in anti-infective investigations [134,135,136]. This review’s goal is to investigate the antibacterial properties of polyphenolic compounds utilizing in vitro assays. Their applications and possible antimicrobial mechanisms are shown in Figure 2 [137,138,139].

Of the most biologically active polyphenols, few of their antibacterial, antiviral, antifungal, etc., properties have been described. Here are a few instances of polyphenols and their listed antimicrobial properties, also reviewed in tabular form [140,141]:

Catechins: These are the types of flavonoids present in cocoa, green tea, and other foods. Catechins have the highest antimicrobial activity against a variety of bacteria, including *Helicobacter pylori*, which is linked to gastritis and peptic ulcer illness, and *Streptococcus mutans*, which causes dental caries. EGC, EGCG, etc. and catechins act as strong antimicrobial agents through DNA gyrase (binding at ATP sites), inhibition of intracellular functions, as well as destruction of biofilm formation in the microbial cells. A slight alteration within the toxin’s configuration causes a reduction in the toxin’s required membrane communications. That, in turn, causes catechin substances to inhibit leukotoxin action. 

We believe that these substances can be utilize internally to cure periodontal diseases and boost patients through improvement therapy protocols. Catechins from tea, such as EGCG, prevent planktonic growth of the bacterium *P. gingivalis* [142,143,144].

Quercetin: Quercetins, having antimicrobial activities, were investigated using the broth dilution technique against: *S. aureus* and *Pseudomonas aeruginosa* ((quercetin = 20 µ/mL); *E. coli* (400 mcg/mL); *Proteus vulgaris* (300 µ/mL); *Shigella flexneri* and *Lactobacillus casei var*. *Shirota* (500 µ/mL)) [101,145,146]. Quercetin shows antifungal activity in literature studies on *C. albicans*. The *C. albicans* strain grew aerobically overnight in 4 mL of Sabouraud broth at 37 °C. After 48 h at 37 °C, viable *Candida albicans* cells were counted, and CFU values were recorded on Sabouraud agar plates, producing around 107 CFU/mL after overnight incubation [147]. The MIC values of quercetin derivative solutions, after 48 h at 37 °C (the minimal amount of flavonoid products that prevent *C. albicans* from growing visibly), were determined to range from 0.06 to 2.50 mg/mL [148].

Resveratrol: This is a stilbene found (with a high content) in red wine and grapes. It has been demonstrated that resveratrol exerts antimicrobial effects on several microorganisms, including *Staphylococcus aureus* and *Helicobacter pylori*. Therefore, in parallel to the biochemical processes stated previously, the main focus of research is to investigate the potential as an inhibitor of the growth of specific harmful microbes like Gram-positive and Gram-negative bacteria, fungi, etc. It could be used in the future to prevent and treat various diseases caused by particular pathogens. *Staphylococcus aureus*, *Bacillus cereus*, *Escherichia coli* ATCC 25922, etc. have been used to test the antimicrobial activities of resveratrol in different settings like agar dilution, microdilution, time–kill curve methods, etc. [149,150,151]. Resveratrol’s antiviral properties are linked to restrictions of viral transmission, the production of proteins, gene transcription, and the generation of nucleic acids, whereas the antioxidant benefit is primarily generated via inhibition of essential genetic routes, such as the nuclear factor-kappa β (NF-κβ) pathway. Resveratrol is beneficial in treating several viral (Epstein–Barr virus (EBV), herpes simplex virus-1 and herpes simplex virus-2 (HSV-1 and HSV-2), as well as respiratory syncytial viral (RPSV), etc.) infections. An anti-inflammatory intermediary by blocking NF-κβ activity, procyclooxygenase-2 activity, and prostaglandin synthesis, in addition to cellular growth at the G1 and G1/S stages, can be observed using resveratrol.

Ellagic acid: The simultaneous use of ellagic acid with other antioxidants, which are well known for their numerous potential antimicrobial benefits, has also been shown to have effective medicinal properties. Antibacterial, antiallergic, antimicrobial, antiviral, and antiparasitic properties were additionally demonstrated for this material [152,153]. Furthermore, ellagic acid has demonstrated immunity toward naturally occurring toxins as well as the toxicity of metals and metalloids. This is a polyphenol found in nuts, berries, and pomegranates. *Streptococcus mutans* and *Helicobacter pylori* are just two of the microorganisms in which ellagic acid has been demonstrated to have an antibacterial effect [154,155,156]. Human rhinoviruses, or HRVs, such as HRV-2, HRV-3, and HRV-4, are the reason for widespread flu and account for millions of dollars’ worth of healthcare expenditures each year. Naturally, ellagic acid (EA) was found to have IC50 values that indicated that it is around 2 times more toxic than ribavirin against HRV-2 (38 μg/mL), HRV-3 (31 μg/mL), and HRV-4 (29 μg/mL). Moreover, 50 μg/mL EA significantly suppresses HRV-4’s RNA synthesis in HeLa cells, according to real-time PCR techniques, indicating that EA blocks viral transformations by focusing on cell molecules instead of virus particles, as well as protecting the cell from the virus.

Curcumin: Curcumin has antimicrobial properties against a variety of bacteria, viruses, fungi, etc. Turmeric contains a polyphenol called curcumin. *Pseudomonas aeruginosa*, *Staphylococcus aureus*, *Escherichia coli*, etc. are strongly inhibited through the destruction of the microorganism’s cell in the presence of curcumin [157,158,159]. Additionally, curcumin has demonstrated strong antibacterial properties towards 65 clinical specimens of *Helicobacter pylori*, with MIC values of 5 and 50 μg/mL, and inhibitory effects toward methicillin-resistant *S. aureus* (MRSA) strains with a minimal inhibitory concentration (MIC) of 125–250 μg/mL. Curcumin’s various bioconjugates, such as di-O-tryptophan-phenylalanine curcumin, di-O-decanoyl curcumin, di-O-palmitoyl curcumin, etc., have demonstrated strong antiviral activity towards a range of viruses, including flock house virus (FHV), parainfluenza virus-3 (PIV-3), feline-infectious peritonitis virus (FIPV), etc. Di-O-decanoyl and di-O tryptophan–phenylalanine curcumins, with half maximal effective concentration (EC_50_) values of 0.011 μM and 0.029 μM, respectively, have demonstrated notable beneficial activity towards FIPV/FHV. On the other hand, in MT-4 cells, bioconjugates showed no appreciable antiviral activity towards HIV-1 (IIIB) and HIV-2 (ROD) varieties [160]. Even so, the strong antibacterial, antioxidant, antifungal, anti-inflammatory, and antiviral properties of curcumin have been proven. However, antimicrobial studies are required for further detailed investigation of more clinical isolates [161,162,163].

Naringenin: Through disruption of DNA replication, cell membranes, and enzyme functions, naringenin demonstrates antibacterial properties against Salmonella typhimurium, Escherichia coli, Pseudomonas aeruginosa, and Staphylococcus aureus. Moreover, it prevents the growth of bacterial biofilms, which may enhance the effectiveness of treatment [164]. Naringenin possesses antifungal properties towards a range of fungi, especially Aspergillus fumigatus, Candida albicans, Cryptococcus neoformans, and dermatophytes like Trichophyton rubrum. These molds are linked to a variety of human infections, such as dermatophytosis (fungal skin infections), systemic fungal infections, and oral thrush. Naringenin fights influenza A and B strains, inhibits HSV-1 and HSV-2, which cause genital and oral herpes, and has antiviral activity against HCV, which causes chronic hepatitis. It also prevents dengue virus replication [165].

Luteolin: Luteolin exhibits antibacterial activity against a range of bacteria, including *Helicobacter pylori*, *Pseudomonas aeruginosa*, *Escherichia coli*, and *Staphylococcus aureus*. Its efficacy is attributed to its mechanisms of action, which include rupturing bacterial cell membranes and blocking bacterial enzymes [166,167]. Numerous fungal pathogens, such as Botrytis cinerea and Penicillium expansum, have been shown to be susceptible to luteolin’s antifungal activity [168]. Luteolin’s antiviral properties and low toxicity were analyzed using LDH and MTT assays. Cell survival trends improved in the presence of PRV, indicating luteolin’s beneficial effects at concentrations below 10 μM. By inducing HO-1 expression and downregulating STAT1/3-dependent NF-κB activation, luteolin suppresses viral inflammation [169,170,171].

**Cyanidin:** It has been used in antibiotics, inhibits *S. aureus* and *K. pneumoniae* microbes about 87% observed by in-vitro method. Elevated levels of cyanidin improve *K. pneumoniae*’s susceptibility to pertinent antibiotics. Generally, synergistic effects are evident in the values of fractional inhibitory concentration (FIC). Antibiotic action is enhanced synergistically by sub-MIC cyanidin [172]. Cyanidin has exhibited antifungal properties against a range of fungal species, including *Botrytis cinerea*, *Candida albicans*, and *Colletotrichum gloeosporioides*. As concentrations are raised, cyanidin-3-glucoside more effectively controls the fungus [173]. Fruit-derived flavonoid cyanidin inhibits the NF-κB and IL-17A pathways to fight disease. In RSV-infected mice, it reduces cytokine levels, inhibits multiple viruses, and minimizes lung damage. It restricts RSV, HSV-1, influenza virus, canine coronavirus, and SARS-CoV-2 multiplication, indicating a wide range of antiviral activity [127].

**Daidzein:** Daidzein is used to treat menopausal symptoms, thrombosis, hypertension, vertigo, and deafness. When it comes to *Staphylococcus aureus*, *Escherichia coli*, *Acinetobacter baumannii*, *Klebsiella pneumoniae*, and *Pseudomonas aeruginosa*, daizenin has a clear antibacterial effect [174,175]. Elicitation causes daidzein prenylation, indicating that interactions between *A. oryzae* and soybeans produce beneficial phytoalexins. Daidzein has also shown significant antifungal activity against *Pyrrhoderma noxium*, *C. lindemuthianum*, and *P. citricarpa* [176]. Preclinical research on daidzein has revealed possible antiviral activity against a range of viruses. This covers viruses like hepatitis C virus (HCV), human immunodeficiency virus (HIV), influenza, etc. Daidzein’s efficacy against these and other viruses varies with its concentration [177,178].

Bioactive polyphenols, which are abundant in nature, are summarized in tabular form to describe their chemical molecular structures, molecular weight (Mol. Wt.), and aqueous solubility (mg/mL), including extracted amounts from different natural sources (Table 2). 

Different polyphenolic compounds were considered for their antimicrobial activity for further use in different applications, such as for medicinal purposes or in food industries. Antibacterial, antifungal, and antiviral studies are mainly considered for checking primarily for further use in formulation processes. Among them, most polyphenols show strong antimicrobial activities. Antibacterial studies have been observed in the literature, including biological applications.

## 6. Determination Assay

### 6.1. Determination of the Antibacterial Activity

Polyphenols cause destabilization or disruption in the cell membrane by combining with the cytoplasmic microbial cell membrane’s lipid bilayer. This breakdown occurs because polyphenolic substances can irreversibly change the structures of cell membranes and interfere with their hydrophobic properties [209]. They can also start spot ruptures that compromise the impermeability of a structure. This produces pores that can be penetrated by cellular material. The physical structure of a cell membrane is distorted when polyphenols are present, which causes the membrane to expand, become structurally unstable, and enhance the material’s inactive penetration [210,211]. The loosening of a microbial cell’s membrane robs it of ATP, ions, and nucleic acids, all of which are necessary for the cell to survive. Certain polyphenols, like flavone-3-ol (tannin), can interfere with the replication of genes required for the synthesis of p-fimbriae proteins. Microbial agents, like bacteria (*Escherichia coli*), are unable to stick to the surface of the epithelium due to a modification in molecular structure. Uropathogenic *E. coli* cannot spread to the surface of the mucilaginous epithelium and subsequently cause a barrier, and they are unable to form an adhesive connection with the receptors found on the host’s cell membranes [212,213]. Several bacterial strains (such as *S. aureus* CNRZ3 [214], *L. monocytogenes* ATCC19115 [215], *P. aeruginosa* ATCC27853 [216], *S. enteritidis* E0220, *B. subtilis* ATCC6633, and E. coli ATCC25922 [214]), are used separately in broth microdilution method to investigate the antibacterial activity of the corresponding polyphenols by cell growth monitoring [217,218]. Mainly, the DMSO solvent was used within a suitable concentration range for antibacterial studies, since solubility of polyphenols are much less or insoluble in aqueous medium. Generally, the buffer could not be used for making the polyphenolic solutions. Antibacterial property shows the percentage of *OD* at a fixed wavelength range of reduction after 24 h at 37 °C, which is computed using the following formula [214,219].

(1) $$BLD=\left[\frac{\left(1-{OD}_{420-580\;oftestwell}\right)}{{OD}_{420-580\;ofcorrespondingcontrolwell}}\right]\times 100$$

where *OD* = optical density (nm), bacteria load difference (*BLD*) = percent reduction of *OD*_420−580_ after 24 h of incubation.

The efficiency of antibacterial drugs on microorganisms is commonly assessed through the use of minimum inhibitory concentration (MIC) and minimum bactericidal concentration (MBC) assays. Associated methods include agar dilution method, microdilution method, E-test (epsilometer test), and broth dilution method. Here, antibacterial activity determination procedures have been stepwise extrapolated through the general disk diffusion process as follows.

First, prepare the nutrient agar plates, which should be prepared using conventional procedures through transferring the liquefied agar onto sterilized Petri plates and letting them set. Bacterial inoculums have to be prepared by choosing which bacterial strains to test. This will depend on the goals, ensuring the turbidity of the bacterial culture is adjusted to meet the 0.5 McFarland standard (about 1.5 × 10^8^ CFU/mL) by suspending it in a sterile solution of saline or broth. (i) After that, if the antibacterial agent is solid, dilute it in a suitable solvent (such as ethanol or DMSO or water) to create a sample solution with a specified concentration. To obtain the appropriate concentrations for testing, dilute the stock solution. (ii) Impregnation of disks: Place sterile filter paper disks onto a sterile surface using sterile forceps. (iii) Then, on each disk, apply a known volume of the antibacterial agent solution. To make sure the disks are completely impregnated, let them air dry. (iv) Evenly smear bacterial suspension across the agar plates’ surface using a sterile cotton swab. To make sure the inoculum is properly absorbed, let the inoculated plates dry for a few minutes. (v) On the surface of the infected agar plates, place the impregnated disks containing the antibacterial agent using sterile forceps. (vi) Incubation: After inverting the plates, incubate them for about 18–24 h at the proper temperature (37 °C) for most bacterial strains [220]. (vii) Zones of inhibition measurement: Using a caliper or ruler, measure the diameter of the clear zones (zones of inhibition) surrounding the disks after incubation. (viii) Note the zone widths for every antimicrobial agent’s strength that was evaluated and analyze the data by calculating the average diameter of the zones of inhibition for each antibacterial agent concentration. (ix) To find the minimum inhibitory concentration (MIC) and evaluate the antibacterial agent’s potency, plot a dose–response curve. Then, determine the tested agent’s antibacterial activity and compare the zone widths and MIC values to published data or standard reference values. Finally, to verify the results, use suitable −ve and +ve controls in the test, such as solvent-only disks and standard antibiotics. Using the technique of disk diffusion, this process offers a fundamental framework for assessing a substance’s antibacterial properties [220,221,222].

### 6.2. Determination of the Antifungal Activity

Identifying an element’s antifungal properties entails evaluating its capacity to prevent the reproduction or survival of fungi. Materials with antifungal action include drugs, natural products, and chemicals. Usually, this is accomplished by using a variety of tests and experimental techniques. The following are some typical procedures and techniques for assessing antifungal activity [223,224,225,226].

First, select the particular fungi toward which the antifungal properties of the selected microorganisms are investigated. They might include experimental strains of various species of fungi, standard strains, or medical isolates. Then comes testing material preparation. If testing a chemical compound or natural extract, prepare the ingredient in various doses. It may be dissolved in the proper solvents to produce a variety of strengths. In this manner and in the future, systems have to be considered for antifungal activities using different methods. Examples are: (i) Agar dilution method: Create several agar plates with progressively higher test material concentrations. Inject the plates with fungus spores. Place the plates in an incubator at the right temperatures for fungi to thrive. The areas surrounding the wells or streaks that hold the drug are the inhibition areas. The stronger the antifungal action, the bigger the inhibition area. (ii) Broth dilution method (minimum inhibitory concentration, MIC): Prepare a series of tubes containing the test material in a growth medium at escalating concentrations. Add fungi spores to each tube. Set the tubes’ temperature to the ideal range for fungus growth. The MIC is the level of the chemical at which visible fungal growth is inhibited. (iii) Disk diffusion method: The filtering disks should be impregnated using the test material. Put the disks on a plate of agar that has been freshly inoculated with fungi. Determine the area of inhibition surrounding the disks after incubating the plate. (iv) Time–kill assay: Use a precise concentration of the experiment’s chemical to the fungus culture. Specimens taken at various times should be plated onto agar to determine the number of growing fungal colonies. This technique reveals how rapidly the chemical eliminates the fungus. (v) Biofilm inhibition assay: Certain molds can create biofilms that become more resistant to antifungal medications. Compounds are evaluated for their impact on biofilm viability and development using particular tests. Analysis of gene expression and quantitative PCR are two methods that can be used to measure how the test drug influences particular fungal genes or pathways.

After that, the cytotoxicity evaluation can be carried out on the test chemical for human or animal cells. When thinking about using the chemical for therapeutic purposes, this is crucial. Finally, using analytical statistics, ascertain whether the detected antifungal properties are statistically important, and run the necessary statistical analysis. The choice of method depends on factors like the nature of the test substance, the fungal species being studied, and the specific research objectives. It is also important to use appropriate controls and replicate experiments to ensure reliable and meaningful results.

Short descriptions of antifungal activity of polyphenols have been extrapolated with several examples. Different fungi survive and make our lives difficult, such as fungal infections caused by *Candida*, *Aspergillus*, *Pneumocystis*, and *Cryptococcus*, the main threatening agents. These can be killed by polyphenolic compounds through the disk diffusion method: daidzein with MIC value 516–1032 µg/mL active on *C. albicans*, genistein active on *T. rubrum* at 1000 µg/mL, naringenin active on *C. graminicola* and *T. deformans* with MIC value of about 3.125 mg/mL, quercetin active on *C. albicans*, *C. glabrata*, and *C. krusei* with MIC value of about 7.8–256 µg/mL, etc. [176].

### 6.3. Determination of Antiviral Activity

Antiviral activities were studied in the following procedure using an in vitro model. Testing substances or compounds to ascertain their efficacy in preventing the replication or spread of viruses is a common step in antiviral activity procedures. This is a basic summary of the steps taken to evaluate antiviral activity [148,165,227].

Selection of virus: (i) Determine which viruses are appropriate for the target infection or illness. (ii) Select a particular strain from the variety of viruses if known.

Culturing of cells: (i) Choose suitable cells for hosting that facilitate the selected virus’s reproduction. (ii) Keep cultivating cells in an environment conducive to the replication of viruses. (iii) Make sure the cells are uncontaminated and in good health.

Test for cytotoxicity: (i) Determine the toxicity of the test material on the host cell population. (ii) Analyses to assess the viability of cells can be carried out through methylthiazolyldiphenyl-tetrazolium bromide (MTT) assay, methoxynitrosulfophenyl-tetrazolium carboxanilide (XTT), or lactate dehydrogenase (LDH) discharge. (iii) Determine a range of concentrations that will not be harmful for upcoming antiviral tests.

Antiviral assay: (i) Introduce the chosen virus into host cells, while varying the test substance’s concentration. (ii) Add the negative controls (without the test material) and positive controls (also referred to as antiviral agents). (iii) Utilizing techniques like quantitative PCR, 50% tissue culture infectious dose (TCID50) assays, or plaque elimination assays, one can quantify the propagation or growth of a virus.

Figure of dose–response: (i) Produce a curve with a dose–response relationship to identify the amount in which the test compound displays antiviral activity. (ii) Determine additional relevant variables, such as the half-maximal inhibitory level (IC_50_).

Studies of time courses and action mechanisms: (i) Evaluate the antiviral activity’s kinetics as influenced by time. (ii) Find the best incubation period to observe the strongest antiviral effects. (ii) Examine the mechanism by which the antiviral effect of the test substance is achieved. (iii) Examine whether it prevents the entry, reproduction, or release of viruses.

Index of selectivity (SI) repeated and verified: (i) By contrasting the cytotoxicity and antiviral activity, determine a selectivity index. (ii) Importantly, particularity for suppressing the virus despite appreciable cytotoxic effects is indicated by a higher SI. (iii) To ensure that results can be repeated, repeat tests. (iii) To verify the wide-spectrum activity, test with various virus strains or kinds of cells.

Reporting, data analysis, and conclusion: (i) Apply statistical analysis to the data. (ii) Describe the findings using IC_50_ values, inhibition percentages, and other pertinent information. (iii) Make judgments regarding the tested substance’s potential for antiviral action.

## 7. Future Prospects

To increase the targeted and controlled release of bioactive polyphenols against microbial organisms, the solubility of polyphenols has to be increased. Modifying the delivery systems having sustained release nature of drug molecules polyphenols strongly protected us from the microorganisms [57,228]. Naturally occurring polyphenols extracted from fruits, leaves, alleges, etc. show lower solubility in aqueous medium, by increasing the solubility of the polyphenols by converting them into the salts can be more useful in different food and biomedical applications [229,230,231]. Using positively charged metals ions, creating a more soluble nature of these polyphenolic compounds through physiochemical modifications can show superior effectiveness against harmful microorganisms as well as upgraded medicinal chemistry [232,233]. It is crucial to keep in mind that the antibacterial, antioxidant, and anticancer activity of these polyphenols can vary based on elements, including the polyphenol’s quantity, solubility in different solvents, the kind of bacterium being targeted, and the tested delivery procedure [234]. A variety of distinct routes may be involved in the mechanisms by which polyphenolic systems perform their antimicrobial properties compared to pure and different modified bioactive polyphenolic substances, which are still under investigation [235,236].

## 8. Conclusions

The necessity to develop novel antimicrobial compounds is ongoing, as the prevalence of antibiotic resistance increases internationally. Phenols display strong antibacterial activity that can be greatly strengthened via modifications into its salt derivatives. They also have a few modular frameworks that enable a wide space of chemical modifications for different applications. *S. aureus* (with methicillin resistance), for example, spreads quickly among patients in healthcare institutions and is thought to be a major contributor to community-associated infections and related deaths. A major healthcare goal is the creation of successful treatment options for resistant microorganisms. Polyphenols are diversely structured substances that have been utilized for centuries to cure illnesses, especially infections. In addition to having antibacterial action, they also have antioxidant, anti-inflammatory, and anticancer properties. Based on available research on the antibacterial properties of polyphenols, they may be suggested as a replacement or supplementary treatment for pathogenic disorders. Consequently, regular and ongoing consumption of polyphenols throughout a lifetime can be described as “bioactive polyphenol schooling”, which explains how repeated and ongoing intake of foreign substances is countered by antimicrobial polyphenol molecules through the development of self-defense mechanisms in the body. Natural polyphenols represent a potentially useful resource of antimicrobial properties, either used independently or in conjunction with currently available medicines to create novel antimicrobial therapeutics and in the food industry.

## Figures and Tables

**Table 2:** Aqueous solubility and extracted amounts from natural sources of a few polyphenols are described in the following table.

Polyphenols	Structures and Mol. Wt. (g/mol)	Solubility in Water (mg/mL)	Group/Sub-group	Amount Extracted from Sources	Natural Sources	Ref.
Ferulic acid	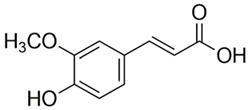 194.18	780	Hydroxylcinnamic acid	0.5–3 mg/g	Bamboo shoot, pineapple, bananas, spinach, and beetroot	[179]
Naringenin	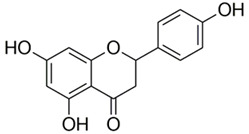 272.25	0.47	Flavanone	~52.03 mg/g	Grapefruits	[164,180]
Baicalein	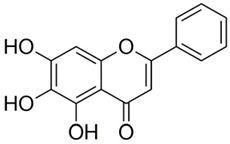 270.237	0.05	Flavone	~10 mg/g	Scutellariagalericulata leaves	[181,182,183]
Rutin	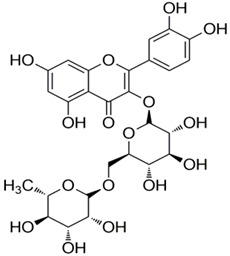 610.517	0.13	Flavonol	~2.7 mg/g	Buckwheat leaf	[184,185]
				0.012–0.484 mg/g	Apples	
Puerarin	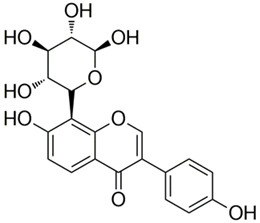 416.38	0.46	Isoflavone	37.51 ± 0.64 mg/g	Root of Pueraria lobata	[186,187]
Quercetin	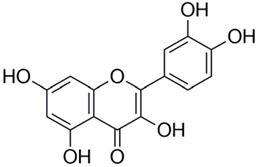 302.236	0.003	Flavonol	0.39 mg/g	Red onions	[188,189,190,191]
				~3.65 mg/g	Capers	
Resveratrol	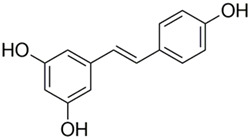 228.247	0.05	Stilbene	1.14–8.69 mg/L	Spanish red grape juice	[192,193]
				~14.3 mg/L	Red wines (global)	
Curcumin	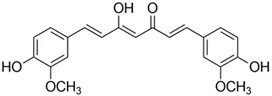 368.39	0.0006	Diarylheptanoid	7.4 mg/g	Lakadong turmeric	[133,194,195]
Secoisolariciresinol	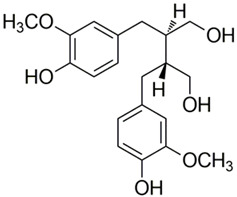 362.422	0.012	Lignan	11.9–25.9 mg/g	Whole flaxseeds	[196,197]
Daidzein	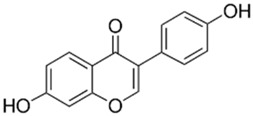 254.23	0.15	Isoflavone	1.2–4.2 mg/g	Soybean	[174,198]
				10–25 mg/g	Red clover	
				0.5–0.6 mg/g	White clover	
Luteolin	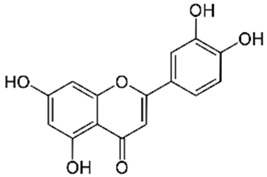 286.24	0.82	Flavone	~37.96 mg/100 g	Radicchio	[166,167,168]
				~34.87 mg/100 g	Raw Chinese celery	
Morin hydrate	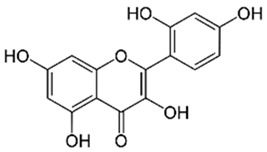 302.236	0.25	Flavonol	--	Figs, sweet chestnut, jackfruit, red wine, seaweed, tea, coffee, and cereal grains	[199]
p-Coumaric Acid	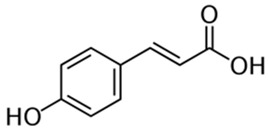 164.16	0.1	Hydroxycinnamic acid	5.77 mg/100 g	Dried fruits, e.g., dates	[200,201]
Caffeic acid	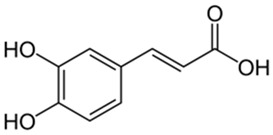 180.16	0.6	Hydroxycinnamic acid	20–22 mg/g	Ceylon cinnamon, star anise, thyme, sage, and spearmint	[202,203]
				2 mg/100 mL	Red wine	
Apigenin	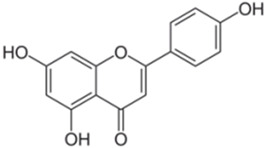 270.24	0.0014	Flavone	78.65 mg/g	Celery seeds	[81,204]
				62.0 mg/g	Spinach	
				45.04 mg/g	Parsley	
Gallic acid	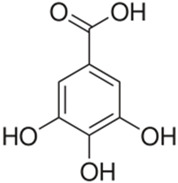 170.12	11.1	Hydroxybenzoic acid	5–309 mg/g 1–15 mg/g	Bearberry leaf, evening primrose	[205,206]
Fisetin	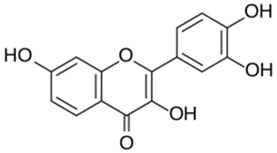 286.24	0.5	Flavonol	160 μg/g	Strawberries	[207,208]
				26.9 μg/g	Apple	
				10.5 μg/g	Persimmon	

## Data Availability

The data can be shared upon request.
